# Transference of multiple resistance to peanut through the development of cross-compatible complex hybrids of wild *Arachis*

**DOI:** 10.1590/1678-4685-GMB-2019-0099

**Published:** 2020-06-08

**Authors:** Alessandra Pereira Fávero, Adriana Regina Custodio, Naiana Barbosa Dinato, Ignácio José de Godoy, José Guillermo Seijo, Marcos Doniseti Michelotto

**Affiliations:** 1 Embrapa Pecuária Sudeste, São Carlos, SP, Brazil.; 2 Embrapa Recursos Genéticos e Biotecnologia, Brasília, DF, Brazil.; 3 Universidade Federal de São Carlos, São Carlos, SP, Brazil.; 4 Instituto Agronómico, Campinas, SP, Brazil.; 5 Universidad Nacional del Nordeste, Instituto de Botánica del Nordeste, Facultad de Ciencias Exactas y Naturales y Agrimensura, Corrientes, Argentina.; 6 Agência Paulista de Tecnologia dos Agronegócios, Polo Centro Norte, Pindorama, SP, Brazil.

**Keywords:** Amphidiploids, wild species, genetic resources, groundnut, Arachis hypogaea

## Abstract

Peanut (*Arachis hypogaea* L.) is a tetraploid species with an A and B genome, while the majority of wild *Arachis* species are diploid with distinct genomes. In pre-breeding programs, one way to introgress interesting wild genes into peanut is by producing amphidiploids. This study aimed at the hybridization between distinct amphidiploids and their characterization, to combine high crossability with peanut, observed in some amphidiploids, with high pest and disease resistances observed in others. These new hybrids were called complex hybrids. Four amphidiploids previously obtained were crossed at four different combinations, and the derived complex hybrids were crossed with four peanut cultivars. Morphological, reproductive, chromosome complement, molecular markers for hybrid identification, phytopatological, and entomological characterizations were performed on the complex hybrids. All cross combinations resulted in complex hybrids. One complete complement of each diploid progenitor was confirmed in each hybrid. Plants of six distinct hybrid combinations were obtained between the complex hybrids and peanut. Based on morphological characterization, differences among progenies from distinct cross combinations were observed. Complex hybrids were considered more resistant to all diseases and pests than peanut cultivars. The simultaneous introgression of genes from four wild *Arachis* species into peanut was possible through the development of complex hybrids.

## Introduction

Peanut (*Arachis hypogaea* L.) is used worldwide, mainly for oil or grain consumption. The cultivated area is concentrated in tropical and subtropical regions, and the world production in 2016 was estimated at 42.22 million tons (USDA, 2016). Although yield averages above 4.3 Tg ha^−1^ are recorded under good management practices, the average yield for most of the peanut-producing countries is only 1.28 Tg ha^−1^ (USDA, 2016). Disease and pest epidemics are leading factors for suppressed yields, and high levels of resistance to many important biotic stresses are not available in the cultivated genepool (Stalker, 2017).

The genetic variability found in wild *Arachis* species is much higher than that found in cultivated peanuts. The genus *Arachis* has 82 recognized species and is divided into nine taxonomic sections (Krapovickas and Gregory, 1994; Valls and Simpson, 2005; Valls *et al.*, 2013, Santana and Valls, 2015; Valls and Simpson, 2017). Section *Arachis* is the most important for peanut breeding, and includes 30 species besides *A. hypogaea* (Krapovickas and Gregory, 1994; Valls and Simpson, 2005). Wild species of this section are diploid (most are 2n=2x=20 and only two 2n=2x=18) except *A. monticola* that is tetraploid like the cultivated peanut (Fernández and Krapovickas, 1994; Lavia, 1998; Peñaloza and Valls, 2005). The cultivated peanut and *A. monticola* are AABB segmental allotetraploids (Fernández and Krapovickas, 1994; Leal-Bertioli *et al.*, 2015). The diploid species were arranged in six different genomes (A, B, D, F, G, and K) according to chromosome morphology, cytogenetic markers, and cross compatibility (Stalker, 1991; Robledo *et al.*, 2009; Robledo and Seijo, 2010; Silvestri *et al.*, 2015).

The difference in ploidy level hinders the direct introgression of genes from wild relatives into the tetraploid peanut, since hybrids are sterile triploids (Simpson, 2001) but the triploid hybrids can be doubled with colchicine and crossed with *A. hypogaea,* followed by self-fertilization generations to recover the 40-chromosomes conditions (Varman, 2001a,b). The best way to introduce wild alleles into peanut is to produce diploid hybrids and to double the chromosome number with colchicine. The synthetic amphidiploids, which can be compatible at different levels with the cultivated peanut are then crossed and backcrossed with *A. hypogaea* (Simpson and Starr, 2001).

The success of introgression of wild alleles, mainly those related to high resistance to pest and diseases, into peanut is not only restricted by the ploidy level barrier, but also by the effective recombination within interspecific or intergenomic hybrids obtained from crosses among more distant species. Different surveys of resistances in *Arachis* showed that the most interesting performances were detected in species that are not closely related to peanut (Stalker *et al.*, 2016; Stalker, 2017). Moreover, the amphidiploids derived from species that are genetically closer to peanut, such as *A. ipaënsis* and *A. duranensis* (Fávero *et al.*, 2005), did not show high resistance against most diseases and pests. By contrast, many of the amphidiploids derived from species that are genetically distant from *A. hypogaea* presented the highest resistances (Michelotto *et al.*, 2016). Therefore, there is a need to combine the high crossability of some amphidiploids with the high resistance of others in complex hybrids for the effective introgression of desirable traits into peanut.

In this context, the goal of the present study was to develop complex amphidiploids that combine multiple high resistances to diseases and pests and the high crossability with *A. hypogaea*. For that purpose, the complex hybrids here developed were characterized by means of morphological, molecular, and cytogenetic markers, by pollen viability analyses, and by assays to evaluate resistance to multiple pests and diseases. Many studies show the importance of introgressing *Arachis* wild genes for pests and diseases resistance, as done in field, laboratory, or greenhouse phenotyping evaluation based on QTL identification (Pande and Rao, 2001; Michelotto *et al.*, 2017; Leal-Bertioli *et al.*, 2015; Zhou *et al.*, 2016). These are the first complex hybrids that include the genome of four distinct species at the same time developed in *Arachis*, some of them were cross-compatible with peanut, and the derived F_2_ showed multiple resistances to pests and fungal diseases.

The results here presented showed that the genomes of four distinct wild species could be used simultaneously for the introgression of alleles into cultivated peanuts. With this approach, it was possible to obtain new complex hybrids and peanut introgressed lines with new interesting allelic combinations for peanut breeding programs.

## Material and Methods

### Development of complex hybrids

The *Arachis* species used for obtaining the amphidiploids are listed in Table 1. All of the *A. hypogaea* accessions used in the introgression crossing are cultivars that are being used by Brazilian producers.

**Table 1:** Accessions of *Arachis* species, collector code, species name, Brazilian accession code (BRA), municipality, state, or country of collection.

Accession*	Species	BRA	Municipality	State/Country**	Genome
GKP 10017	*A. cardenasii* Krapov. & W. C.Gregory	013404	Roboré	BOL	AA
VNvEv 14167	*A. duranensis* Krapov. & W. C.Gregory	036200	Salta	ARG	AA
VSGr 6389	*A. gregoryi* C.E. Simpson, Krapov. & Valls	012696	Vila Bela da Ssa. Trindade	MT	BB
VSGr 6325	*A. helodes* Martius ex Krapov. & Rigoni	012505	S. Antonio do Leverger	MT	AA
KG 30006	*A. hoehnei* Krapov. & W. C. Gregory	036226	Corumbá	MS	K?
cv. IAC Tatu ST	*A. hypogaea L.*	011606		BRA	AABB
cv. IAC Runner	*A. hypogaea L.*	037389		BRA	AABB
cv. IAC Caiapó	*A. hypogaea L.*	037371		BRA	AABB
cv. BR 1	*A. hypogaea L.*	033383		BRA	AABB
KGBPScS 30076	*A. ipaënsis* Krapov. & W. C. Gregory	036234	Ipá	BOL	BB
VPoBi 9401	*A. linearifolia* Valls & C. E.Simpson	022608	S. Antonio do Leverger	MT	AA

* Collector/Institutional abbreviations: B= Banks; Bi= L.B. Bianchetti; Ev= A. Echeverry; G= W.C. Gregory; Gr= A. Gripp; K= A. Krapovickas; Nv= L. Novara; P= J.R. Pietrarelli; Po= A. Pott; S= C.E. Simpson; Sc= A. Schinini; V=J.F.M. Valls.

** Country or state: ARG= Argentina; BOL= Bolívia; BRA=Brazil; MT= Mato Grosso; MS= Mato Grosso do Sul; SP= São Paulo.

Crosses were performed at Embrapa Recursos Genéticos e Biotecnologia, Brazil, from January to May 2005 under greenhouse conditions. Emasculations were carried out in late afternoon and pollination was done in the next early morning.

Four previously obtained synthetic amphidiploids (Fávero *et al.*, 2006, 2015) were used in this study (Table 2). Crosses involving four different hybrid combinations were performed: (K 30076 x V 14167)^4x^ x (K 30006 x V 6325)^4x^; (K 30076 x V 14167)^4x^ x (V 6389 x V 9401)^4x^; (V 6389 x V 9401)^4x^ x (K 30006 x V 6325)^4x^; (V 6389 x V 9401)^4x^ x (K 30006 x G 10017)^4x^. After harvest, seeds were dried and stored in cold chambers (10 °C/35% RH) until the next growing season.

**Table 2:** Amphidiploids used in crosses as female and male parents, number of pollinations (NP), number of hybrids obtained (H) and percentage of success in hybrids (PS), percentage of stained pollen (PC), number of F_2_ seeds obtained (F_2_) and genome.

Code	Female parent		Male parent	NP	H	PS	PC	F_2_	Genome
HC1	(K 30076 x V14167)^4X^	x	(V 6389 x V 9401)^4X^	554	19	3.43	65.13 a	34	B_i_B_g_A_d_A_l_
HC2	(K 30076 x V14167)^4X^	x	(K 30006 x V 6325)^4X^	296	2	0.68	0.33 c	1	B_i_A_d_K?_ho_A_he_
HC3	(V 6389 x V 9401)^4X^	x	(K 30006 x V 6325)^4X^	416	4	0.96	12.50 b	0	B_g_A_l_K?_ho_A_he_
HC4	(V 6389 x V 9401)^4X^	x	(K 30006 x G 10017)^4X^	330	12	3.64	7.25 c	0	B_g_A_l_K?_ho_A_car_
	Total			1,596	37				

### Development of hybrids between complex hybrids and peanut

The F_1_ complex hybrids here obtained were crossed with four cultivars of *A. hypogaea* (*A. hypogaea* subsp. *fastigiata* var. *fastigiata* cv. IAC-Tatu-ST and cv. BR-1, *A. hypogaea* subsp. *hypogaea* var. *hypogaea* cv. IAC-Runner 866 and *A. hypogaea* cv. IAC-Caiapó) (Table 3).

**Table 3:** *Arachis hypogaea* cultivars and complex hybrids used in crosses as female and male parents respectively, number of pollinations (NP), number of hybrids obtained (H) and percentage of success in hybrids (PS), percentage of stained pollen (PC) and number of F_2_ seeds obtained (F_2_).

Female parent		Male parent	NP	H	PS	PC	F_2_
IAC Caiapó	x	HC1	134	1	0.74	65.34	8
IAC Runner	x	HC1	87	1	1.15	80.69	18
IAC Tatu ST	x	HC1	146	0	0	Na	0
BR 1	x	HC1	184	0	0	Na	0
IAC Caiapó	x	HC2	20	0	0	Na	0
IAC Runner	x	HC2	38	2	5.26	89.42	44
IAC Tatu ST	x	HC2	53	1	1.89	76.35	87
BR 1	x	HC2	48	1	2.08	Ne	0
IAC Caiapó	x	HC3	74	0	0	Na	0
IAC Runner	x	HC3	52	0	0	Na	0
IAC Tatu ST	x	HC3	157	0	0	Na	0
BR 1	x	HC3	91	0	0	Na	0
IAC Caiapó	x	HC4	57	0	0	Na	0
IAC Runner	x	HC4	36	0	0	Na	0
IAC Tatu ST	x	HC4	194	0	0	Na	0
BR 1	x	HC4	117	0	0	Na	0
Total			1,488	6			

Ne= non-evaluated

Na= not applicable

### Morphological characterization of the complex hybrids

Twenty five morphological characteristics were evaluated in the main axis, in lateral branches, and in the flowers of plants kept under greenhouse conditions. Leaflet descriptors were measured in the first expanded leaves, in four replications of each genotype. The morphological descriptors of the main axis (MA) and lateral branch (LB) were: length and width of the apical and basal leaflets, length of petiole and petiolule, length and width of the stipule fused portion, length of the stipule free portion. The morphological descriptors of the flowers (F) were: length and width of the standard and wing, hypanthium length, length of the posterior and inferior lips. The measurements were taken in millimeters with a digital caliper. Data were analyzed using the analysis of variance and Tukey test and based on Principal Component Analysis.

### Reproductive characterization of complex hybrids

Pollen viability estimations were performed by staining with 2% glycerol-acetic carmine. Four flowers were collected from each plant and 200 pollen grains were counted per flower. Data were analyzed by analysis of variance and Tukey test.

### Identification of hybrids using SSR markers

Progenies and parents were analyzed by microsatellite markers (Table 4) developed for *A. hypogaea* (Moretzsohn *et al.*, 2013). Total genomic DNA was extracted from young and fresh leaflets of 41 genotypes, including parents and progenies individuals, according to the method of Doyle and Doyle (1990). The amount and quality of the DNA were evaluated by 1% agarose gel electrophoresis. PCR assays were run with 0.2 μl DNA *Taq* polymerase (5 U/μL), 0.5 μL buffer (with Mg), 1.0 μL dNTPS (2.5 nM), 1.0 μL ultrapure water, 1.2 μL BSA (2.5 mg/mL), 0.1 μL of each primer (10 μm) and 2.0 μL genomic DNA, in a final volume of 6 μL. Amplification reactions were performed in an ABI 9700 (Applied Biosystems, Foster City, CA, USA) thermal cycler, under the following conditions: 94 °C for 5 min, followed by 30 cycles at 94 °C for 1 min, 58 °C for 1 min (depending on annealing temperature of the primer), 72 °C for 1 min, and final extension at 72 °C for 1 min. The allelic detection of 30 SSR loci was performed in an ABI377 automated sequencer in a multiplex loci system (Table 4). Genetic diversity was analyzed by the PowerMarker V 3.25 and NTSYS programs.

**Table 4:** Multiplex systems, labeled primers and their respective fluorescence, base pair size, amplification temperature, and the products that were amplified and analyzed.

Multiplex	Primer	Fluorescence	Size (bp)	Temperature (°C)	Analyzed
1	TC3E02	Blue	270-310	58	X
	AC2H11	Green	230-270	58	X
	TC7G10	Blue	110-142	58	X
2	TC7H11	Blue	340-360	58	X
	RN2C06	Green	190-220	58	X
	TC6E01	Blue	154-186	58	X
3	TC7A02	Blue	308-320	58	X
	GI-338	Green	240-270	58	X
	TC4F12	Blue	220-232	58	X
	GI-832	Green	200-210	56	X
4	TC11A02	Green	284-292	58	X
	TC6H03	Blue	210-228	58	X
	RN22G07	Green	180-210	58	X
5	TC9F10	Green	286-320	56	X
	TC1D02	Blue	242-278	56	X
	GI-342	Green	210-240	58	X
6	RNO-681	Green	310-350	54	
	TC7E04	Blue	290-300	56	X
	TC9F04	Green	122-142	54	
7	AC2B03	Green	296-308	54	X
	TC2B09	Blue	190-200	52	X
	RI1F06	Green	312-372	56	X
8	GI-1107	Green	360-384	52	X
	TC1A02	Blue	240-276	54	X
9	TC3H02	Blue	280-300	54	X
	TC11A04	Green	172-204	52	X
	TC6G09	Blue	132-146	50	X
10	TC2A02	Blue	194-212	48	X
	RNO-615	Green	390-400	56	
11	TC1E01	Blue	154-248	48	X
	TC9C12	Green	256-300	54	
	TC7C06	Blue	148-176	52	X
	TC11H06	Green	190-214	52	X
12	TC1A01	Blue	202-222	54	
	TC2D06	Blue	196-224	48	
	TC3E05	Blue	358-370	48	
	RN8C09	Green	260-290	56	X

### Identification of chromosome complements in the complex hybrids

The presence of the chromosome complements of each diploid species in the complex hybrid nuclei was investigated by the detection of chromosome markers that included morphology of some chromosome pairs (A9 and SAT chromosomes), heterochromatin amount and distribution, and the number and localization of 18-26 rDNA and 5S rDNA (Robledo *et al.*, 2009; Robledo and Seijo, 2010). For chromosome preparations, root apices pretreated with 2 mM 8-hydroxyquinoline for 3 h and fixed in 3:1 absolute ethanol:glacial acetic acid (Fernández and Krapovickas, 1994) were digested in 1% (w/v) cellulose (Onozuka) plus 10% (v/v) pectinase (Sigma) solution in 0.01 at 37 °C for 2 h. The meristematic cells were squashed in 45% acetic acid.

The 18S–26S and 5S rDNA loci were localized using probes isolated from genomic DNA of *A. hypogaea* (Robledo and Seijo, 2008). Pretreatment of preparations, chromosome and probe denaturation, conditions for the *in situ* hybridization (hybridization mixes contained DNA probes at a concentration of 2.5 – 3.5 ng/L, with a stringency to allow sequences with 80 – 85% identity to remain hybridized), posthybridization washing, blocking and indirect detection with fluorochrome-conjugated antibodies were performed according to Moscone *et al.* (1996) and Seijo *et al.* (2004). Chromosomes were analyzed and photographed with an epifluorescence microscope equipped with a digital camera system. Red, green and blue images were captured in black and white using appropriate filters for TRITC, FITC, and DAPI excitation, respectively. Digital images were combined and then processed for color balance, brightness, and contrast for uniformity across the image.

### Phytopathological and entomological characterization of complex hybrids under laboratory conditions

Bioassays were performed using detached leaves (Moraes and Salgado, 1982) under controlled laboratory conditions to verify resistance to rust (*Puccinia arachidis* Speg.), fall armyworm (*Spodoptera frugiperda* J.E. Smith), and velvetbean caterpillar (*Anticarsia gemmatalis* Hübner). The four complex hybrids (three in the velvetbean caterpillar assay) and the IAC Tatu ST peanut cultivar as susceptible control were included in the assays.

#### Characterization of complex hybrids for resistance to rust (Puccinia arachidis Speg.)

Four leaves of each genotype were evaluated after 23 days of experiment. The bioassay was carried out in Petri dishes filled with a cotton layer and one blotter paper according to Moraes and Salgado (1982). The inoculation was performed using a spore solution at 100,000 spores of rust mL^−1^. The number of pustules per leaf area (cm^2^) was counted. Data were analyzed using the *t*-test.

#### Characterization of complex hybrids for resistance to velvetbean caterpillar (Anticarsia gemmatalis Hübner)

One leaf of each genotype and two caterpillars (first- or third-instar) were kept in each sealed Petri dish filled with a cotton layer and one blotter paper (Moraes and Salgado, 1982). Four replications per genotype using first-instar caterpillars were evaluated seven days after assembling the trial. The third-instar caterpillars were evaluated after four days. Data of damaged leaf area were analyzed by the *t*-test.

#### Characterization of complex hybrids for the resistance to fall armyworm (Spodoptera frugiperda J.E. Smith)

One leaf of each genotype and two first-instar caterpillars were kept in a sealed Petri dish filled with a cotton layer and one blotter paper. Four replications of each genotype were analyzed after a five-day experiment. The damaged leaf area was evaluated by a 1-4 damage scale (1-resistant, 2-moderate resistant, 3-moderate susceptible, 4-susceptible). Data of damaged leaf area were analyzed by the *t*-test.

### Phytopathological characterization under field conditions

#### Characterization of the complex hybrids

Field trials were carried out at APTA Polo Centro Norte in Pindorama, São Paulo State, Brazil (21º13’ S and 48º55’ W), where inoculum pressure for peanut phytopathogenic fungi is considered high. Three complex hybrids were evaluated: HC1 ((K 30076 x V14167)^4x^ x (K 30006 x V 6325)^4x^; HC3 (V 6389 x V 9401)^4x^ x (K 30006 x V 6325)^4x^; HC4 (V 6389 x V 9401)^4x^ x (K 30006 x G 10017)^4x^) and the peanut cultivar IAC Caiapó. For the *Sphaceloma arachidis* assay, the peanut cultivar BR-1 was also included as control.

The resistance trial was performed in four randomized blocks, with five plants per 1.5 meter rows with 0.90 spacing between rows. Seeds and seedlings were planted in pots and transplanted to the field when rooted. The evaluation was performed at 90 days. Two types of evaluation were performed: 1) using a 1-9 score scale that identifies defoliation index and damaged leaf area (Subrahmanyam *et al.*, 1982), and 2) disease severity in the most attacked leaf of the plant. The most damaged leaves of each plant and each genotype were collected for evaluation. The evaluated diseases were late leaf spot (*Cercosporidium personatum* Berk and M.A. Curtis), rust, early leaf spot (*Cercospora arachidicola* Hori), and scab (*Sphaceloma arachidis* Bitanic and Jenkins). Leaves were scanned and evaluated by the analysis of the damaged area using the Image Tool^®^ Free Software. Statistical analyses were performed using the *t*-test.

#### Characterization of the F_2_ progenies for foliar fungal diseases

The field assay was performed including F_2_ progenies, amphidiploids, complex hybrids, and *A. hypogaea* cultivars. Seeds were treated with Plantacol^®^ fungicide (10 g per 100 kg seeds) and put to germinate into blotter paper, conditioned at 26 ? 3 °C, 70 ? 10% RH, and photoperiod of 12 hours. Seedlings were put in plastic cups (200 ml) with soil and manure (3:1) and kept in greenhouse conditions. Fifteen days after emergence, the plants were put in field. Plants of the F_1_ were planted by branches. Genotypes were placed as random blocks with four replications, with five plants per plot (1 m between plants, 1.5 m between plots and 1.8 m between rows). All plots were fertilized with 8-28-16 NPK formula as 250 kg/ha dosis. The insecticide tiametoxam + lambda-cialotrina (Engeo^TM^ Pleno, Syngenta) was sprayed every 15 days at 0,15 L/ha dosis to thrips (*Enneothrips flavens* (Moulton, 1941) (Thysanoptera: Thripidae)) and rednecked armyworm (*Stegasta bosquella* (Chambers, 1875) (Lepidoptera: Gelechidae) control. Pre-emergent trifluraline herbicide (2.5 L/ha) was used for weed control. Manual weeding control were done as necessary.

Foliar disease resistance evaluations were performed at 65, 80, 95 and 125 days after the transplant to the field. A 1 to 9 diagrammatic scale was used, where 1 meant no symptoms and 9 meant high disease infestation and high defoliation (Subrahmanyam *et al.*, 1982). The severity was evaluated by the use of the area under disease progress curve (AUDPC) based on the formula AUDPC = Σ [((y1 + y2)/2)*(t2 - t1)], where y1 and y2 are two consecutive evaluations performed on times t1 and t2, respectively. A principal component analysis was performed based on AUDPC and the detached leaves data.

## Results

### Development and reproductive behavior of complex hybrids

A total of 1,596 pollinations were performed, resulting in hybrids from all the combinations, with a total of 37 individuals considered as complex hybrids (Table 2). The hybridization rate ranged from 0.68 to 3.64%. Hybrids were conserved in pots under greenhouse conditions. The percentage of stained pollen grains of the complex hybrids ranged from 0.33 to 65.13% (Table 2).

**Figure 1:**
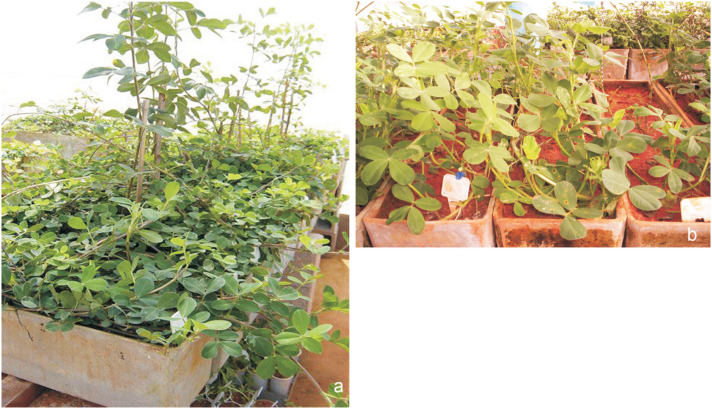
Complex *Arachis* hybrds (a) Complex hybrid (*A. ipaënsis* x *A. duranensis*)^4x^ x (*A. gregoryi* x *A. linearifolia*)^4x^, (b) cv. IAC Runner x (*A. ipaënsis* x *A. duranensis*)^4x^ x (*A. gregoryi* x *A. linearifolia*)^4x^.

Only two combinations produced fertile hybrids: HC1 (*A. ipaënsis* x *A. duranensis*)^4x^ x (*A. gregoryi* x *A. linearifolia*)^4x^ (Figure 1a) and HC2 (*A. ipaënsis* x *A. duranensis*)^4x^ x (*A. hoehnei* x *A. helodes*)^4x^. The first combination produced 34 F_2_ seeds, while the second one generated only one F_2_ seed (Table 2). The combinations HC3 (*A. gregoryi* x *A. linearifolia*)^4x^ x (*A. hoehnei* x *A. helodes)* ^4x^ and HC4 (*A. gregoryi* x *A. linearifolia*)^4x^ x (*A. hoehnei* x *A. cardenasii)* ^4x^ generated F_1_ hybrids with higher pollen viability than HC2, but did not produce F_2_ seeds.

### Morphological characterization of the complex hybrids

The morphological characterization showed significant differences in nine out of the 25 descriptors analyzed in the complex hybrids (Table 5). These descriptors were: length and width of the basal leaflet, and width of the apical leaflet in the main axis (MA); length of the apical leaflet and length of the stipule adnate portion in the lateral branch (LB); standard and hypanthium length, and length and width of the wing in flowers (F). The coefficients of variation among descriptors ranged from 5.9 (wing length) to 41.13% (length of the stipule adnate portion on the main axis).

**Table 5:** Morphological descriptors of complex hybrids.

Descriptor	HC1	HC2	HC3	HC4	CV%
Apical leaflet length MA*	63.39 a	62.12 a	67.55 a	48.56 a	15.51
Basal leaflet length MA	57.20 ab	53.68 ab	61.93 a	45.18 b	13.94
Apical leaflet width MA	31.29 a	26.19 ab	24.66 ab	20.64 b	16.08
Basal leaflet width MA	24.59 a	20.16 ab	19.40 ab	16.52 b	17.49
Petiolule length MA	19.21 a	17.45 a	20.63 a	15.48 a	17.41
Petiole length MA	53.37 a	51.98 a	59.11 a	53.82 a	17.25
Length of the stipule adnate part MA	6.02 a	11.39 a	5.85 a	7.43 a	41.13
Length of the stipule free part MA	30.50 a	27.39 a	31.72 a	26.23 a	11.89
Width of the stipule adnate part MA	3.63 a	3.61 a	3.54 a	3.83 a	21.51
Apical leaflet length LB	34.01 ab	45.69 a	33.02 ab	30.34 b	20.15
Basal leaflet length LB	31.34 a	36.81 a	28.63 a	27.79 a	23.44
Apical leaflet width LB	25.17 a	26.48 a	19.90 a	18.10 a	20.94
Basal leaflet width LB	19.60 a	20.75 a	15.46 a	15.26 a	22.76
Petiolule length LB	22.73 a	28.22 a	18.22 a	23.89 a	30.26
Petiole length LB	12.55 a	13.16 a	12.67 a	10.51 a	17.47
Length of the stipule adnate part LB	5.91 ab	7.74 a	4.11 b	4.37 b	17.04
Length of the stipule free part LB	20.18 a	21.41 a	18.42 a	15.61 a	15.58
Width of the stipule adnate part LB	4.32 a	3.70 a	3.74 a	3.97 a	17.14
Standard length	10.07 b	13.20 a	12.20 a	12.26 a	5.97
Standard width	6.54 a	7.75 a	6.83 a	6.77 a	8.97
Wing length	7.44 b	8.34 ab	9.08 a	9.08 a	5.90
Width of the wing width	4.91 b	6.38 ab	5.82 ab	6.43 a	11.54
Inferior lip length	6.99 a	6.69 a	8.88 a	9.14 a	13.82
Posterior lip length	5.57 a	5.74 a	5.59 a	6.51 a	15.52
Hypanthium length	13.40 b	28.50 a	29.76 a	34.44 a	22.29

* in mm. MA = Main axis, LB = Lateral branch. CV% = coefficient of variation (in percentage)

Data with the same letters were considered similar at 5% probability

**Figure 2:**
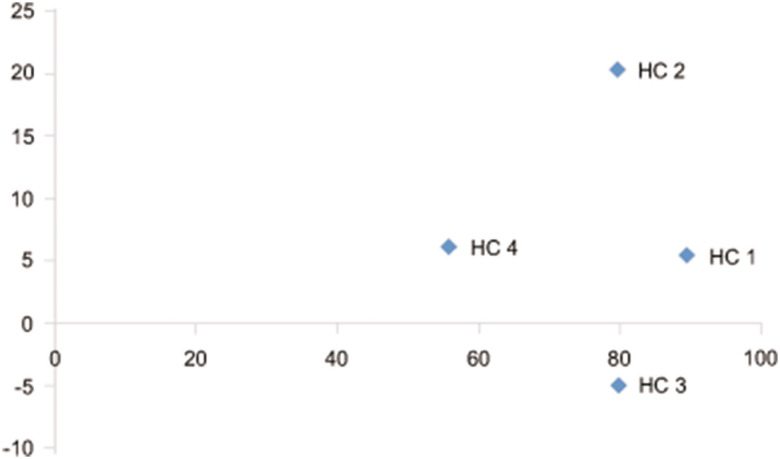
Principal Component Analysis based on morphological data of the complex hybrids. The plot represents the spatial distribution of the hybrids according to the first two axes.

Eigenvalues showed that the two first components explain 82.23% of the total morphological variation. The eight main descriptors that discriminated the complex hybrids in the principal component analysis were (in order of importance): apical leaflet length, basal leaflet length, apical leaflet width, and basal leaflet width of the main axis; apical leaflet width, length of the stipule free portion, and basal leaflet length of lateral branches, and finally, length of the stipule free portion on the main axis. The dispersion observed in Figure 2 evidenced a clear morphological distinctness among the complex hybrids, being HC1 and HC3 the most similar.

### Mitotic chromosomes of F_1_ complex hybrids

**Figure 3:**
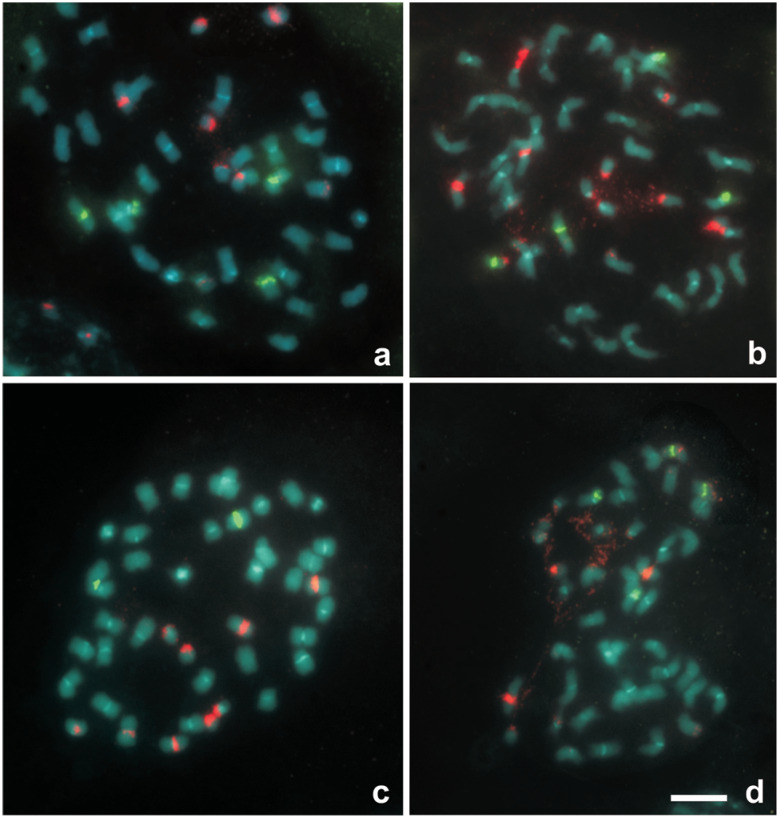
Representative somatic metaphases of the four complex hybrids after double fluorescent *in situ* hybridization (FISH), showing yellow-green FITC signals from the 5S rDNA probe, and red TRITC signals from the 18S-26S rDNA probe. DAPI counterstaining (light blue) was used to highlight the heterochromatic bands and to stain euchromatin. (a) HC1; (b) HC2; (c) HC3; (d) HC4. In all the tetraploids, the 18S–26S loci with extended secondary constrictions were those of the A10 pair and more rarely those of the A2 pair or those of the B10 pair. Scale bar = 5 μm.

All the complex amphidiploid hybrids analyzed presented 2n=4*x*=40. The cytological markers evidenced that the amphidiploids are composed of one complete chromosome complement of each of the diploid progenitors used in the initial crosses. The genome constitutions of the amphidiploids were as expected (Table 6); while HC2 (Figure 3b), HC3 (Figure 3c), and HC4 (Figure 3d) were AA K?hoB. The complements of the B genome (*A. ipaënsis* and *A. gregoryi*) were clearly detected by the absence of conspicuous heterochromatic centromeric bands. The complements of the A genome (*A. cardenasii, A. duranensis, A. linearifolia*) were distinguished by the presence of conspicuous heterochromatic centromeric bands in all their chromosomes and by the A9 pair, which is the smallest chromosome with the largest heterochromatic band (around 40% of the chromosome length) and diffuse chromosome arms. The complement of *A. hoehnei* was also detected by the presence of heterochromatic bands in all their chromosomes, and the presence of a small chromosome but structurally different from the A9 (without diffuse arms). The patterns of 18-26S and 5S rDNA of *A. ipaënsis* and *A. gregoryi* were conserved in the complex hybrids. Most of the chromosome markers here analyzed revealed similar patterns in all the of A genome diploid species, thus the identification of species-specific chromosomes in the amphidiploids was not possible or tentative. However, the number of rDNA loci and A9 chromosomes and the pattern of heterochromatin observed was as expected in HC1, HC2 and HC4. Only in HC3 the number of observed 5S rDNA was two instead of the four expected from their parental species.

**Table 6:** Chromosome markers observed in the complex hybrids. Expected markers were summarized according to published data.

Hybrid	45 S rDNA		5 S		A9 + small *A. hoehnei*	
	Expected	Observed	Expected	Observed	Expected	Observed
HC1	8 (3ipa+2greg) + (2dur+1lin)	8 (5B +3A)	4 (1ipa+1greg) + (1dur+1lin)	4	2	2
HC2	10 (3ipa)+(2dur+2 hoeh+3hel)	10(3B+4-5A+2ho)	4 (1ipa)+(1dur+1hoeh+1hel)	4	2+1	2+1
HC3	8 (2greg)+(1lin+2hoeh+3hel)	8 (2B+3A+2ho)	4(1greg)+(1lin+1hoeh+1hel)	2 (4)	2+1	2+1
HC4	9 (2greg)+(1lin+2hoeh+4card)	9 (2-3B+5A+2ho)	4(1Greg)+(1lin+1hoeh+1card)	4	2+1	2+1

The analysis of secondary constrictions and patters of 18-26S rDNA revealed the occurrence of amphiplasty. In most cases, the extended nucleolar organizing regions observed in late prometaphase or metaphases were in chromosomes that belong to the A genome.

### Obtaining hybrids between complex hybrids and cultivated peanuts

After 1,488 hybridizations performed in 16 different cross combinations, only six hybrid individuals were obtained (Table 3): two from crosses between cultivars of *A. hypogaea* subsp. *hypogaea* var. *hypogaea* (IAC Caiapó and IAC Runner) and HC1 (*A. ipaënsis* x *A. duranensis*)^4x^ x (*A. gregoryi* x *A. linearifolia*)^4x^ (Figure 1b), and four hybrids between three cultivars of *A. hypogaea* (IAC Runner, IAC Tatu ST and BR 1) and the complex hybrid HC2 (*A. ipaënsis* x *A. duranensis*)^4x^ x (*A. hoehnei* x *A. helodes*)^4x^.

The percentage of stained pollen grains of F_1_ hybrids between the complex hybrids and *A. hypogaea* was relatively high and varied from 65.34 to 89.42 (Table 3). Notably, the percentages of stained pollen grains were higher with the HC2 hybrid in which the genome formula was BK?hoAA than with HC1, which had the genome formula BBAA. The F_1_ hybrids obtained from the crosses of cv. IAC Caiapó and IAC Runner 886 with HC1 produced 8 and 18 F_2_ seeds, respectively. The F_1_ hybrids generated from the crosses of cv. IAC Runner 886 and IAC Tatu ST with HC2 produced 44 and 87 F_2_ seeds, respectively.

### Identification of hybrids via molecular characterization

Molecular markers were informative for the identification of hybrid individuals (Table 7). Plants considered hybrid on morphological and reproductive analysis presented the expected bands inherited from their respective male parents (in gray). The markers TC7A02 and TC6E0 were the most informative for the hybrid identification. Although the AC2H11 and RN2C06 markers were less informative, they also contributed to corroborate the results of the two former microsatellites.

**Table 7:** Polymorphic microsatellite markers used to identify complex hybrids of *Arachis.* In the genotypes column, the materials are arranged in groups of three (or four) rows, indicating the female (F) and male (M) parents and subsequently the hybrid between these parents tested. Gray colored cells show the alleles shared between the male parent and its hybrid(s).

Genotypes		TC7A02		RN2C06		TC6E01				AC2H11		
		a1	a2	a1	a2	a1	a2	a3	a4	a1	a2	a3
F	(K 30076 x V14167)^4x^	269	305	200			160	186		213	221	235
M	(V6389 x V9401)^4x^	261		200		158	190	210			221	
H	HC1	273	305	200		160	188	210			221	235
F	(K 30076 x V14167)^4x^	269	305	200		160	186			213	221	235
M	(K 30006 x V 6325)^4x^	265		200		192	208			221		
H	HC2	265	299	200		160	186	192	208	221	235	
F	(V6389 x V9401)^4x^	261		200		158	190	210		221		
M	(K 30006 x V 6325)^4x^	265		200		192	208			221		
H	HC3	263	273	200		148	190	208		221		
F	(V6389 x V9401)^4x^	261		200		158	190	210		221		
M	(K 30006 x G 10017)^4x^	265		204		206	220			213		
H	HC4	265	273	200	204	210	220			221		
F	IAC-Tatu-ST	289	299	188	200	160	202			221	251	
M	HC1	273	305	200		160	188	210		221	235	
H	IAC-Tatu-ST x HC1	263	299	200		160	186	194		221		
F	IAC-Tatu-ST	289	299	188	200	160	202			221	251	
M	HC2	265	299	200		160	186	192	208	221	235	
H	IAC-Tatu-ST x HC2	265	305	200		160				221		
F	IAC-Tatu-ST	289	299	188	200	160	202			221	251	
M	HC3	263	273	200		148	190	208		221		
H	IAC-Tatu-ST x HC3	289	297	188	200	160	166	202		221	251	
H	IAC-Tatu-ST x HC3	289	297	200		160	202			221	251	
F	IAC-Caiapó	291	299	188	200	160	180			221	249	
M	HC1	273	305	200		160	188	210		221	235	
H	IAC-Caiapó x HC1	289	299	200		160	180	188		221	249	
F	IAC-Caiapó	291	299	188	200	160	180			221	249	
M	HC2	265	299	200		160	186	192	208	221	235	
H	IAC-Caiapó x HC2	291	299	188	200	160	180			221	249	
F	IAC-Caiapó	291	299	188	200	160	180			221	249	
M	HC4	265	273	200	204	210	220			221		
H	IAC-Caiapó x HC4	291	299	188	200	148	160	180		221	249	
F	IAC-Runner 886	289	299	188	200	160	194			221		
M	HC1	273	305	200		160	188	210		221	235	
H	IAC-Runner 886 x HC1	261	299	200		160	188	194		221	251	
F	IAC-Runner 886	289	299	188	200	160	194			221		
M	HC2	265	299	200		160	186	192	208	221	235	
H	IAC-Runner 886 x HC2	269	299	200		160	186	194		221	235	251
H	IAC-Runner 886 x HC2	269	303	200		148	160	186	194	221	235	251
F	BR1	289	297	188	200	160	200			221	251	
M	HC2	265	299	200		160	186	192	208	221	235	
H	BR1 x HC2	273	297	200		160	186	200		221	235	251

a = allele Gray data means same alleles between the male parent and the hybrid

### Phytopathological and entomological characterization of complex hybrids under laboratory conditions

All the complex hybrids showed significant higher resistance to rust, fall armyworm, and velvetbean caterpillar when compared to the control IAC Tatu ST (Table 8). The resistance to caterpillars showed a similar pattern in the two instars analyzed.

**Table 8:** Mean damaged area caused by *Puccinia arachidis, Anticarsia gemmatalis* in the first- and third-instar and degree of damage caused by *Spodoptera frugiperda* in the first instar in *Arachis* complex hybrids and *Arachis hypogaea* cv. IAC Tatu ST.

Genotypes	*P. arachidis*	*A. gemmatalis*		*S. frugiperda*
		1^st^ instar	3^rd^ instar	Leaf damage
HC1	0.0000**	1.2650**	1.7784**	2**
HC2	0.0000**	NE	NE	2**
HC3	0.0000**	0.5344**	7.0589**	1**
HC4	0.0144**	0.0280**	5.8682**	2*
IAC Tatu ST	0.2715	26.5860	20.8090	4

** Significant difference between genotype and control at 1% probability.

* Significant difference between genotype and control at 5% probability.

NE - not evaluated

**Figure 4:**
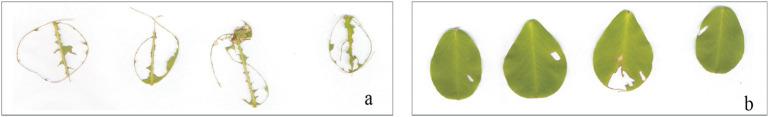
Evaluation of the damaged leaf area. (a) in the cultivar IAC Tatu ST; (b) in the complex hybrid (*Arachis ipaënsis* KG 30076 x *Arachis duranensis*V 14167) x (*Arachis gregoryi* V 6389 x *Arachis linearifolia*V 9401) after four days of inoculation of the velvetbean, *Anticarsia gemmatalis* in the 1^st^ instar.

Besides the lower degree of lesions observed in the hybrids compared with the *A. hypogaea* cultivar analyzed (Figure 4), lesser growth of fall armyworm was also observed when they were fed on complex hybrids leaves.

### Phytopathological characterization under field conditions

#### Characterization of the complex hybrids

The main disease observed in the field evaluations was the late leaf spot, although lesions caused by other pathogens were also observed in different degrees. Table 9 shows that all the complex hybrids analyzed proved to be more resistant than the cultivar IAC Caiapó to late leaf spot, rust, and early leaf spot. Complex hybrids were also more resistant to scab than cultivar BR 1.

**Table 9:** Evaluation of complex hybrids (HC) and *Arachis hypogaea* cv. IAC Caiapó for resistance to *Puccinia arachidis* (Pa), *Cercosporidium personatum* (Cp), *Cercospora arachidicola* (Ca), *Sphaceloma arachidis* (Sa) and total damaged area by foliar fungal diseases.

Genotypes	Pa	Cp	Ca	Sa	Damaged area
HC1	1**	3**	1**	1**	0.0139**
HC3	1**	2**	2**	1**	0.0142**
HC4	1**	3**	2**	1**	0.0097**
BR1	NE	NE	NE	8	NE
IAC Caiapó	4	5	4	NE	0.0658

** Significant difference between genotype and control at 1% probability.

All diseases scored from 1 to 9. NE- non-evaluated.

### Characterization of the F_2_ progenies

**Figure 5:**
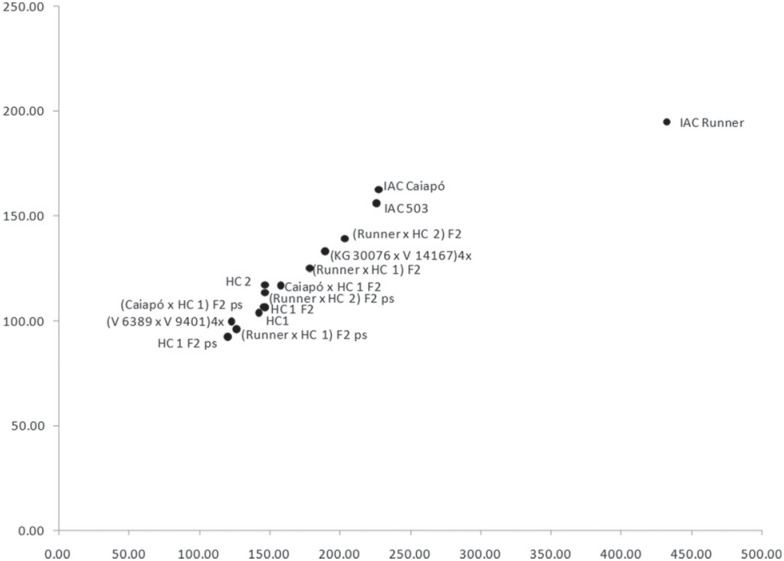
Biplot graph based on AUDPC and detached leaves evaluation data from parents, F_1_ progenies, F_2_ progenies averages and selected plant of each F_2_ progeny (sp).

A biplot graph (Figure 5) was performed based on the AUDPC and the detached leaves data from parents, averages of F_2_ progenies and the outstand plant of each progeny (selected plant - sp). All F_2_ hybrids were located closer to the wild parents than to *A. hypogaea* cultivars. All peanut cultivars, even the most resistant one (IAC 503), were more susceptible than any wild parents, the F_1_ and F_2_ hybrid progenies.

## Discussion

It is known that the amphidiploid (*A. ipaënsis* x *A. duranensis*)^4x^ shows the best cross compatibility with peanut, but it is not the most resistant to diseases and pests (Michelotto *et al.*, 2015, 2016). This is because *A. ipaënsis* and *A. duranensis* are the ancestors of cultivated peanut (Kochkert *et al.*, 1996; Seijo *et al.*, 2004; Fávero *et al.*, 2006; Bertioli *et al.*, 2016). The high crossability of this AABB wild amphidiploid is highly relevant, since it can be used as a bridge for introgression of resistance genes located in other wild species that produced non-crossing amphidiploids, or which generate sterile F_1_ population with the cultivated peanut. Here, we demonstrated that the introgression of genes from non directly related diploid species into peanut is feasible by using a bridge AABB amphidiploid.

The hybridization assays between amphidiploids demonstrated that not all the combinations are equally compatible, since two of the four F_1_ complex amphidiploids produced viable F_2_ seeds. Moreover, it is worthy of note that the F_2_ of HC1 was more fertile (with 34 F_2_ seeds) than that of HC2 (with only one F_2_ seed). This difference between HC1 and HC2 is probably related to the genome constitution of the male amphidiploid. In HC1, *A. gregory* and *A. linearifolia* belong to the A and B genomes, respectively, and therefore a high chromosome homeologous pairing is expected in the F_1_ meiosis with A. *ipaënsis* (B genome) and *A. duranensis* (A genome). In HC2, while *A. helodes* is a well-known A genome species, *A. hoehnei* does not belong to the B genome, being probably of the K genome (Custodio *et al.*, 2013). Even though it was demonstrated that the B and K genomes have partial homeology (Leal-Bertioli *et al.*, 2015), it is expected that meiosis was not as regular in the case of AABB complex hybrids as discussed above. The difference in meiotic behavior discussed here is clearly reflected in the pollen stainability of the F_1_ of these hybrids, (65.13 in the F_1_ of HC1 and 0.33% in that of HC2). The sterility of F_1_ complex hybrids HC3 and HC4 demonstrated that even among species within the same genome there are significant different reproductive isolation barriers preventing production of viable F_2_.

Our results also showed a differential reproductive behavior of HC1 [(*A. ipaënsis* x *A. duranensis*)^4x^ x (*A. gregoryi* x *A. linearifolia*)^4x^] and HC2 [(*A. ipaënsis* x *A. duranensis*)^4x^ x (*A. hoehnei* x *A. helodes*)^4x^] with peanut. Interesting is the fact that in both cases the female amphidiploid came from the hybridization of the diploid progenitors of peanut. It is worthy of note that the F_1_ hybrids obtained showed more than 60% of stained pollen and produced fertile F_2_, which indirectly evidenced a good homeologous pairing between the chromosomes coming from the diploid species with those of each subgenome (A and B) present in peanut. This aspect is crucial for the transmission of desirable characters from wild diploids into the peanuts subgenomes.

It is worth to mention the importance of the genetic base broadening for peanut obtained by crosses between five distinct diploid species. The most remarkable antecedent is the introgression of resistance to *Meloidogyne arenaria* (Neal) Chitwood and *M. javanica* (Treub) Chitwood in the peanut cultivar COAN. As the inheritance of this character is considered as a single, dominant gene (Bendezu and Starr, 2003), it was possible to release the first cultivar that presented a gene located in wild *Arachis* species and transferred to *A. hypogaea* (Simpson and Starr, 2001). The incorporation of genes from wild species of *Arachis* to *A. hypogaea,* in addition to representing a broadening of the genetic base, has contributed to the reduction of production costs, since the introduction of these genes contributes to decrease the incidence of diseases and the use of pesticides, thus generating great savings for the producer (Stalker, 2017).

Hybridization and polyploidy usually have been reported as processes that induce genomic and epigenetic rearrangements (Chen, 2007; Madlung and Wendel, 2013). Only few allopolyploids remain as examples that have not undergone conspicuous chromosome rearrangements, among them is *A. hypogaea* (Seijo *et al.*, 2018). The sum of chromosome markers here analyzed by FISH revealed that the complex amphidiploids showed a high stability in their karyotypes. *Arachis hoehnei* needs particular attention. Although it was traditionally assumed to belong to the B genome *sensu lato* because it lacks the A9 pair (Fernández and Krapovickas, 1994), the presence of large heterochromatic bands in its karyotype demonstrates that it may not belong to the B genome species. Therefore, although the genome constitution of this species still has to be determined, the accession used here may not be considered as belonging to the B genome as defined by Robledo and Seijo (2010). From a cytological point of view it may be better placed among the K genome species.

Concerning the morphological characterization of the germplasm here analyzed, our data demonstrated that the first two principal components explained a high percentage (> 80%) of the total variance, and that nine characters standout as important in the phenotypic discrimination. This is in complete accordance with previously published results using simple amphidiploids (Fávero *et al.*, 2015; Paula *et al.*, 2017). The fact that the hybrids were morphologically more similar to the amphidiploid progenitors than to the parent *A. hypogaea* (except for the hybrid IAC 503 x (*A. gregoryi* V 6389 x *A. stenosperma* V 12488)^4x^) evidenced a high percentage of wild alleles in the progenies, supporting a significant broadening of the peanut gene pool to be used for breeding.

Leaf pests and diseases are among the most important factors that limit the economically sustainable production of peanuts worldwide. Late leaf spot and rust, if not controlled, can cause decreases of up to 70% in the production and affect speanut quality (Michelotto *et al.*, 2013). The two peanut cultivars used in the present study were chosen because cv. IAC Caiapó is considered the most resistant cultivar to the late leaf spot and rust in the market, but susceptible to the early leaf spot; and cv. BR 1 is susceptible to scab. Despite the partial resistance in IAC Caiapó, all the interspecific hybrids were more resistant than the *A. hypogaea* genotypes included in both the assay done under laboratory and field conditions. Our study confirms that the resistance to these fungi present in wild diploids (Fávero *et al.*, 2009) can be introgressed into peanut and, eventually, sources of resistance from different species can be pyramided in elite peanut varieties.

The evaluation of damaged leaf area due to foliar fungal diseases aims at the observation of how much the leaf can be attacked by foliar fungi, regardless of the pathogen. The evaluation was done by the total damaged leaf area. According to data reported by Fávero *et al.* (2005), in natural infestation under greenhouse conditions greater resistance to late leaf spot and rust was observed in amphidiploid and segregating individuals than in cultivated peanut. In agreement with studies on resistance to leaf spot and rust, resistance to these diseases is polygenic, complex, and probably controlled by recessive genes (Dwivedi *et al.*, 2002; Mace *et al.*, 2006; Leal-Bertioli *et al.*, 2010).

Due to the susceptibility to pests, such as thrips (*Enneothrips flavens* Moulton) and the rednecked peanut worm (*Stegasta bosquella* (Chambers), peanut production can be severely decreased. This susceptibility is one of the main peanut crop limitations (Lourenção *et al.*, 2007). The use of insect-resistant peanut cultivars may have important benefits, as they keep the pest below the economic damage levels, avoid environment pollution, and reduce the chemical control costs (Lara, 1991). The bioassays here performed, using detached leaves under laboratory conditions to verify the complex hybrids resistance to fall armyworm and velvetbean caterpillar, comparing the complex hybrids with the peanut cultivar IAC Tatu ST, revealed a significant reduction in the damaged leaf area. Moreover, reduction in the growth rate of armyworm caterpillar, when they were fed with complex hybrids leaves, indicates antibiosis resistance. Campos *et al.* (2011) also verified this type of resistance in some peanut cultivars, but with lower intensity. According to Di Bello (2015), the runner peanut cultivars IAC 147 and IAC Runner 886 have antibiosis resistance that affects the larval survival of *S. bosquella*.

To conclude, it was possible to introgress wild alleles into peanut from non closely related wild diploid species (*A. gregoryi, A. helodes*, and *A. hoehnei*) by the production of complex hybrids. We demonstrate that it is feasible to introgress genes from distant wild species using complex hybrid developed from a cross between one peanut compatible amphidiploid with another one made by crossing more distant wild species.
